# Exploring nurses’ perspectives on patient safety culture in neonatal intensive care units: a phenomenological study

**DOI:** 10.1186/s12912-025-03937-6

**Published:** 2025-10-15

**Authors:** Khulud Essa Hadi, Seham Mansour Alyousef, Sami Abdulrahman Alhamidi, Maha Abdulaziz Alonazi, Hanan Atallah Alotaibi, Shahad Mohammed Jaafari, Juliana Linnette D’Sa

**Affiliations:** College of Nursing, King Saud University, Riyadh, Saudi Arabia

**Keywords:** Patient safety culture, Neonatal intensive care units, Qualitative content analysis

## Abstract

**Background:**

The neonatal intensive care unit (NICU) is a stressful environment that makes it challenging for neonatal nurses to adhere to safe care for neonates. However, little research has focused on explaining patient safety culture (PSC) in the NICU. Therefore, this study used in-depth qualitative methods to explore the concept of PSC and its dimensions from the perspectives of nurses working in the NICU.

**Methods:**

This study used a phenomenological descriptive qualitative design. Data was collected through in-depth semi-structured interviews with 15 NICU nurses working in Riyadh, Kingdom of Saudi Arabia. The participants were selected through purposive sampling, and the data were analysed using the deductive approach.

**Results:**

Analysis of the interviews revealed 10 main themes and 33 sub-themes related to the concept of patient safety culture in the NICU. Main themes, such as communication, teamwork, effective handoffs and transitions, and positive leadership, had a positive impact on patient outcomes and minimizing risks. Alternately, inadequate staffing and workload were seen to have a negative impact on patient safety and quality of care.

**Conclusion:**

The findings revealed systemic barriers (staffing shortages, workload, communication gaps) and facilitators (teamwork, leadership support, peer mentoring). Furthermore, brings out emotional and ethical dimensions of safety such as fear of blame, moral distress when unable to deliver optimal care). Moreover, offers practical recommendations for tailored interventions in NICUs that involve education, training, teamwork, implementing daily huddles, continuous practice, encouraging open reporting and non-punitive safety climates, all of theses lead to safety enhancement.

## Introduction

Patient safety culture (PSC) is clearly a key aspect of patient care. Accordingly, the World Health Organization (WHO) has organized a plan to fight problems such as disabilities and death from unsafe care [1]. Data provided by the WHO has shown that unsafe care has led to the loss of 64 million disability-adjusted life years, and is the most common cause of disability and death.

According to the International Council of Nursing (ICN), patient safety is the foundation of quality care [2]. As a result, health system management has viewed strengthening.

PSC as a significant measure to improve nursing care quality [3].

Safety culture has been defined as individual and group beliefs, perceptions, attitudes, competencies, and patterns of behavior that shape the dedication and capabilities of healthcare organizations [4]. However, limited research has focused on explaining PSC in the NICU [5].

The NICU is a stressful environment that makes it challenging for neonatal nurses to adhere to safe care for infants [6]. The weakness and vulnerability of neonates encourage nurses and other healthcare providers to maintain a strong safety culture [7]. Therefore, to achieve high-quality care, nurses must implement a safety culture that diminishes such harmful impacts [8].

National and international studies have shown that in the most comprehensive definition, safety culture includes issues in several dimensions, such as “Overall perceptions of patient safety”, “Frequency of events reported”, “Communication and openness”, “Manager expectations and actions promoting patient safety”, “Organizational learning”, “Teamwork within units”, “Feedback and communication about error”, “Non-punitive response to errors”, “Staffing”, “Management support for patient safety”, “Teamwork across units”, and “Handoffs and transitions” [9]. The Hospital Survey on Patient Safety Culture (HSOPSC) questionnaire was created as a result of the combination of these factors, and it is the most reliable instrument for evaluating the safety culture from the viewpoint of the staff [10, 11]. Safety culture in NICUs has received little attention, and there is, therefore, a shortage of information explaining safety culture in these units [6]. In addition, the safety culture in NICUs differs from that of other units due to the unique care provided and the high vulnerability of premature infants. Despite extensive local and global research using various quantitative methods, there is still no clear consensus on the definition of safety culture and its link to quality of care [12]. Given the multiple dimensions of safety culture and the unique NICU environment, quantitative studies alone are insufficient to explain this complex concept [13]. Therefore, the current study was conducted using in-depth qualitative methods more appropriate for gaining access to deeper facets of safety culture from the perspectives of nurses in the NICU.

## Theoretical framework

This study used the Donabedian Model of Quality Care as a theoretical framework. The model focuses on three key dimensions: structure, process, and outcomes. The structural dimension applies the organizational and environmental factors that affect the delivery of care and patient safety. In the context of this qualitative study, which involves staffing levels, adequate nurse-to-patient ratios are required to ensure that nurses can deliver cautious and attentive care [14]. Furthermore, training and education has revealed that continuous professional development and training in safety protocols enhance nurse competence and confidence in managing complex cases [15].

The process dimension consists of the interactions between healthcare providers and patients, as well as the protocols and procedures followed in delivering care. For instance, open and effective communication among healthcare team members is important to achieving a culture of safety [16]. This includes handoff processes, interdisciplinary rounds, and feedback mechanisms. Moreover, teamwork is a collaborative practice among nurses and other healthcare professionals that promotes an achievement environment in which safety problems can be handled quickly [17].

Finally, the outcome dimension examines the impact of care processes on patient safety and quality of care. This involves patient safety activities such as monitoring and evaluating adverse events, near misses, and sentinel events in order to explore patterns and adopt preventive actions [9]. Furthermore, a positive safety culture not only improves patient outcomes but also boosts work satisfaction and retention among nursing personnel, particularly in high-stress environments like the NICUs [18].

## Literature review

Various international research projects have explored the complexities of fostering a robust PSC within NICUs. These high-acuity environments bring unique obstacles, including professional personnel shortages, lack of communication, and hierarchical dynamics, which can all negatively affect the quality and safety of neonatal care. For instance, a study in Brazil examined PSC among multidisciplinary teams in NICUs, indicating ‘organizational learning-continuous development’ and ‘teamwork’ as strengths, while ‘non-punitive response to error’ and’staffing’ were identified as opportunities for improvement [19].

A previous study conducted a systematic review and meta-analysis to investigate the level of PSC among nurses working in hospitals around the world. Out of 1,507 initial records, 21 studies met the inclusion criteria, resulting in a total sample size of 10,951 participants [20]. The authors reported an overall PSC score of 3.341 (95% CI: 3.221–3.460), indicating a moderate safety culture among hospital nursing personnel worldwide. Within the PSC aspects assessed, “Teamwork within units” received the highest mean score (3.719; 95% CI: 3.594–3.844), indicating a high level of collaboration and communication within nursing teams. “Staffing” had the lowest mean score (3.096; 95% CI: 2.980–3.212), indicating that staffing shortages and workload constraints are continuous challenges affecting patient safety [20].

In Saudi Arabia, a retrospective analysis evaluated trends in the Hospital Survey on Patient Safety Culture (HSOPSC) across three cycles (2019, 2021, and 2022). It highlighted persistent challenges such as weak management support, which led to a prevalent blame culture and stagnation in safety-event reporting [21, 22]. On the other hand, organizational learning, continuous improvement, and patient safety ratings decreased over time, as well as the lowest-scoring composites were staffing and response to error [21]. Furthermore, no domain reached the 75th percentile in positive response rates [21, 22]. Adverse event data released by the Saudi Ministry of Health (MoH) from 2012 to 2015 revealed that 91% of these incidents were deemed preventable [22]. In light of this concern, Saudi Arabia has embarked on initiatives to improve PSC such as the Saudi Patient Safety Center (SPSC) represents one of the MOH’s efforts to foster better healthcare on a national scale [22].

However, a thorough understanding of PSC across different continents is still insufficient, which hampers the ability to recognize regional patterns and effective practices that could improve PSC on a local and global scale [22, 23].

To enhance PSC in NICUs, it is crucial to address these issues through interventions tailored to the specific context, qualitative explorations into nurses’ experiences and perceptions [24].

In addition, existing studies have primarily concentrated on general hospital settings or associated neonatal care topics. For instance, research conducted in the Eastern Province examined the issue of missed nursing care in NICUs, highlighting factors such as workload, staffing, and communication obstacles, yet it did not specifically assess PSC [25].

Other investigations in Saudi Arabia have evaluated PSC in more expansive hospital environments, including King Abdullah Medical City in Makkah, utilizing the HSOPSC survey [26] as well as in intensive care units and tertiary hospitals [27, 28]. Together, these results point to a notable gap in understanding how neonatal nurses and NICU personnel in Saudi Arabia perceive patient safety culture, indicating a vital area for further research [29] Accordingly, the research questions addressed in this study are as follows:

1. How do nurses perceive patient safety in NICUs?
2. What dimensions of PSC influence patient safety from nurses’ experience in (NICUs)?
3. What are the patient safety issues that face nurses in NICUs?
4. What are the strategies from the nurses’ perspectives to enhance the PSC in NICUs?

## Methods

### Study design

A phenomenological approach was employed to understand the perceptions of nurses which is frequently used to explore participants’ perceptions and experiences with specific phenomena pertaining to nursing and other healthcare fields [30]. As a result, this study design helps the researcher explain nurses’ perspectives regarding patient safety culture in NICUs.

### Setting

The study was conducted in one tertiary care hospital in the central region of the Kingdom of Saudi Arabia. The hospital has one NICU with 44 neonatal beds/cots and 106 staff nurses.

### Participants

15 registered nurses working in the NICUs and providing direct care to the neonates were invited to participate in the study. The inclusion criteria were nurses who had previous working experience in the NICU for a minimum of one year. Nurses with less than a year’s experience in NICU and who were not fluent in the English language were excluded. All of the participants were female, including three Saudi nurses and twelve non-Saudi nurses. Participants included staff nurses, charge nurses, and head nurses.

Purposive sampling was used in recruiting the participants. Purposive sampling is often used in qualitative research to allow the selection of a specific group of persons or units for analysis, according to the aim and objectives of the study [31].

### Data collection

Fifteen registered nurses were interviewed by the first author (Kulud Essa Hadi) from March 8 to March 20, 2024. The authors met with the head nurse of the NICU and discussed the study objectives and the plan to conduct semi-structured interviews. Furthermore, the researcher explained the voluntary nature of participation, anonymity and confidentiality of the data, and their right to withdraw from the study without any consequences. The head nurse encouraged sixteen registered nurses to conduct interviews during break time, but one of whom declined because she was busy with patients. The fifteen nurses who agreed to participate were interviewed.

The participants agreed to audio-record the interview and signed the informed consent form. The face-to-face interviews were conducted in a quiet room in the NICU, ensuring privacy. The interview consists of 16 questions were guided by using the HSOPSC questionnaire, developed by the Agency for Healthcare Research and Quality (AHRQ) in 2019 (Table 1) [9]. All interviews were conducted in English and started with an introduction and lasted between 40 and 60 min.

One-by-one in-depth semi-structured interviews with participants were conducted, a format which is more effective than other interview styles because it enables researchers to get comprehensive data and supporting documentation from participants while keeping the study objectives in mind [32]. All interviews were audiotaped and transcribed.

In qualitative studies, sampling continues until data saturation [33]. In this study, although data saturation was achieved after interviewing nine participants, six more interviews were applied to further ensure data saturation.

**Table 1:** Interview guide based on the HSOPSC questionnaire

Questions	Dimension of HSOPSC
(1) What does neonatal safety, in your opinion, mean? When you hear the term “safety”, what comes to mind? What determines the safety of neonates?	Overall perceptions of patient safety
(2) How did it affect the patient when you or one of your colleagues made a mistake in the unit? In such situations, how does the unit manage errors?	Frequency of events reported
(3) In your interaction with your partners, what factors allow you to discuss an error with each other if it occurs?	Communication and openness
(4) How will you know if one of your partners makes an error? What happened after you reported an error to yourself or one of your teammates?	Feedback and communication about errors
(5) How do the hospital’s president and unit manager promote neonatal safety? What is the impact?	Management support for patient safety
(6) Based on your experience, have you or your partners ever been concerned about the implications of reporting an error? Can you provide an example?	Non-punitive response to errors
(7) How do you think concerns like excessive workloads and staff shortages in the unit affect neonatal safety?	Staffing
(8) How does teamwork affect newborn safety in your unit? (9) How can you motivate yourself and your teammates to work together in your unit?	Teamwork within units
(10) How do you believe the interaction between your unit and other wards in the hospital affects newborn safety?	Teamwork across units
(11) How does your unit or the hospital help you learn from your own or your teammates ' errors? (12) From your experience, what are the best methods to learn from the errors made by you or your partners?	Organizational learning
(13) Has neonatal safety ever been risked during a shift change, a transfer to another ward, or during admission? Can you tell me more details about that?	Handoffs and transitions
(14) How do you think the actions of hospital administrators, such as the president, matron, head nurse, or ward director, affect neonatal safety? (15) How do management’ expectations of you affect neonatal safety? (16) Based on your experience, what are the best strategies that managers use to promote patient safety in your unit?	Manager expectations and actions promoting patient safety

### Data analysis

The transcribing software MAXQDA 24 was used to manage data. The data in this study were analysed using the qualitative content analysis method [34]. To prepare for analysis, all recorded data was transcribed verbatim. The researchers then looked through the data several times to become acquainted with its content. This helped to comprehend the general context and intricacies of the responses. Transcript data were arranged in Microsoft Word tables with separate columns for participant identifiers, responses, and analytic memos, enabling structured organization and ease of thematic analysis. The codes were organized into themes and sub-themes via ongoing comparison. The researchers methodically examined each theme, investigating how it related to the research questions and current literature. This entailed analysing the meanings and implications of the themes. The next phase was to write up the findings, which included a full description of the topics accompanied by statements from participants. The table concluded with a discussion of the implications for practice and future research.

### Trustworthiness

Credibility, dependability, confirmability and transferability are four standards identified by [35] that were included in the current research to ensure the trustworthiness of the data. To improve the credibility of the study, a pilot study was employed to analyse the coded interviews to ensure procedural integrity so that interviewers or observers could practice and get feedback before starting the main study. Furthermore, it might allow the authors to notice if participants misunderstood a question, or if a setting wasn’t ideal for collecting good data [36]. Moreover, focusing on long-term interaction and continuous observation, a lot of time was spent on collecting data; when the interview text and extracted codes were presented to the participants, they commented on their accuracy. In addition, if there were any ambiguous points in the data analysis, a phone interview was conducted to clarify the ambiguities. To ensure the dependability of the findings, the researchers sought the advice and supervision of experts. The researchers attempted to prevent confirmation bias when determining conformability by not supporting their hypothesis in data interpretation and not eliminating any results that contradicted their opinion. In addition, to maximize the transferability of the findings, a sample with maximal variety in terms of age, experience, and educational level was employed.

### Ethical considerations

Ethical approval was first obtained from the ethics committee of the King Saud University and a tertiary care hospital in the central region of the Kingdom of Saudi Arabia with reference number KSU-HE-25-138.

Participants received information about the entire research project, including the procedure and aims. Written informed consent was obtained. Participants’ anonymity was maintained by not including their names in any documents. Moreover, confidentiality was maintained throughout the research. All audio recordings were handled only by the researchers, were password-protected.

## Results

Fifteen registered nurses in the NICU were interviewed. At the end of the data collection and analysis, a total of 10 main themes and 33 sub-themes were determined, which include professional and organizational dimensions that explain the PSC from the perspectives of neonatal nurses.

### Main theme 1: Perceptions of patient safety

#### Sub-theme 1: Continuous care

The findings indicated that patient safety in neonatal care is primarily understood in terms of the fragility of newborns and the need for continuous, specialized care. Participants highlighted that neonates are extremely vulnerable due to their small size and delicate health, necessitating constant attention and careful handling. Therefore, a key aspect of neonatal safety is ensuring that newborns receive round-the-clock monitoring and support. This includes maintaining stability in their condition and promptly addressing any medical needs.

*It means our babies are very fragile*,* very small. We want to take all of them with 24-hour care*,* continuity care. (Nurse 1).*

#### Sub-theme 2: Infection control

Infection control emerged as a critical component of patient safety, as neonates have underdeveloped immune systems and are highly susceptible to infections. Participants emphasized the importance of safe practices to prevent infections.

*We must prevent infection for the baby by safe practice. (Nurse 2).*

#### Sub-theme 3: Adherence to medical protocols and policies

The participants explained that adherence to medical protocols was a fundamental aspect of ensuring patient safety. Following established guidelines and best practices in neonatal care helps reduce risks and improves outcomes for newborns.

*No errors means you will do all care according to the protocols*,* according to the policies. It is considered as a fundamental aspect of care. (Nurse 3).*

#### Sub-theme 4: Alertness

The participants defined neonatal safety as ensuring the baby is safe from harm in all aspects, including medication administration and environmental safety. Furthermore, it is a comprehensive approach to safety by emphasizing the need to be cautious and alert in all aspects of neonatal care.

*… safe care and care for the baby. For example*,* safe medication administration and a safe environment for the baby also. To be safe*,* no harm need to be cautious and alert*,* for everything surrounding the baby. (Nurse4).*

Overall, patient safety in neonatal care is perceived as a multifaceted concept that integrates continuous care, infection control, alertness, and strict adherence to medical protocols, all of which are essential for protecting fragile newborns.

### Main theme 2: Events reported

#### Sub theme1: Error reporting system

The findings highlighted a structured and proactive approach to error management within the neonatal unit. The participants described an error reporting system that ensures any incidents or mistakes are formally documented. This systematic approach allows for transparency and accountability in patient care.

*I will report the event first to the manager*,* and we are reporting to the system that we have (Nurse 5).*

#### Sub-theme 2: Quality team in Follow-up

According to participants, a crucial component of the reporting system is the role of the quality team in follow-up. Once an error is reported, the quality team is responsible for investigating the incident and implementing necessary improvements to prevent a recurrence. This follow-up process ensures that reported issues lead to actionable changes in practice.

*I will report it first. And the way we are reporting system we have. Then the quality team will come and follow up this one for improvement. (Nurse 5).*

#### Sub-theme 3: Non-punitive approach to error management

The participants noted that the neonatal unit follows a non-punitive approach to error management, emphasizing learning rather than blame. Staff members are encouraged to report errors without fear of punishment, fostering a culture of safety and openness. This approach helps identify systemic issues and improve patient care without discouraging staff from acknowledging mistakes.

“*We discuss this. We should not do it again and be more keen and observant.” (Focus on improvement rather than blame.) (Nurse 6).*

*I feel like respect and not judging and trying to help. (Nurse 7).*

#### Sub-theme 4: Learning and improvement from errors

Furthermore, the participants acknowledged learning and improvement from errors is a key principle guiding the unit’s practices. Errors are viewed as opportunities for growth, leading to protocol revisions and better training for healthcare providers.

*If there is an error that can cause the patient anything*,* I will inform my senior… I will not judge or blame; I will try to help… just to make her comfortable that you will not be negative. … it’s for your improvement. (Nurse 3).*

#### Sub-theme 5: Continuous monitoring and feedback mechanisms

The participants clarified that the unit ensures continuous monitoring and feedback mechanisms, reinforcing a culture of ongoing quality improvement. Regular assessments and feedback loops help maintain high standards of care, addressing any gaps in practice as they arise.

*“They do for her a full course… how to do medication administration.” (This training was discussed and shared with the team for collective learning.) (Nurse 9).*

Overall, the neonatal unit’s approach to error management is structured, non-punitive, and focused on continuous learning, ensuring that patient safety remains a top priority.

### Main theme 3: Communication and openness

#### Sub-theme 1: Verbal communication and collaborations

The participants considered that nurses should use verbal communication, huddles, and unit-wide discussions to address safety concerns.

*Communicate with us verbally*,* and the whole unit should participate in making them alert either with other nurses or with the head nurse*,* and work as a team. So good communication with your unit team will help you improve patient safety. (Nurse 8).*

### Main theme 4: Teamwork within unit

#### Sub-theme 1: Double-checking system

The participant clarified that procedures need to be double-checked in the unit. Therefore, nurses rely on each other to verify overall care.

*Teamwork is very important*,* especially in a unit*,* because we need to double-check everything. It’s a high risk for the patient*,* even for the smallest thing. So the teamwork it’s good. This will keep the patient safety really high in our unit. (Nurse 3).*

#### Sub-theme 2: Proper communication and orientation

The participants discussed that teamwork is crucial in ensuring neonatal safety, especially in critical cases. Furthermore, proper communication and orientation for new staff and clear communication help maintain high standards of care.

*Proper communication and then for the new staff proper orientation of what we are supposed to do here in the NICU. (Nurse 2).*

### Main theme 5: Teamwork across the units

#### Sub-theme 1: Mutual support

The participant noted that there is coordination between units (e.g., NICU and paediatric intensive care units (PICUs). Therefore, the nurses should ensure smooth patient transfers by guiding incoming staff from other units and adapting to different workflows. Furthermore, sharing knowledge across units is very important, so that nurses from different departments learn from each other’s experiences and practices to enhance patient care.

*Because we have pull-in and pull-out from PICU. So helping each other*,* especially if the PICU is pulling in our unit*,* there are maybe some differences between how the unit*,* how the NICU work system*,* and the PICU work system. So we should assure them*,* guide them*,* let them feel safe. If they need anything*,* they should ask because they are helping us in our work also. (Nurse 10).*

#### Sub-theme 2: Orientation

The participant clarified that a structured approach, such as staff orientation, ensures the smooth integration of staff from other departments. A one-week orientation is provided to familiarize them with NICU protocols, ensuring they can safely care for neonates.

*The impact is about pulling up stuff*,* like moving stuff from the other department to the NICU*,* giving them one week here for orientation so they can provide care for our neonates. (Nurse 4).*

### Main theme 6: Workload and staffing

#### Sub-theme 1: Burnout and fatigue

Participants noted that high workload and staff shortages can lead to burnout, reducing the quality of care.

*With work overload and with the duties that we have*,* it will really affect us because you have to be prepared each time you go on duty. If you are burned out*,* you are tired. You cannot give the proper care for your neonates. (Nurse 11).*

### Main theme 7: Role of hospital leadership

#### Sub-theme 1: Managerial oversight

The participant mentioned that leaders conduct regular rounds, check on medications and documentation, and ensure adherence to safety protocols.

*The leaders are always making rounds and checking*,* for the patients*,* for the medications*,* for the care*,* asking about the patient itself*,* making sure from the system*,* from the documentation we are doing. This is helping us. If we missed anything*,* they will tell us; if there is any mistake*,* they will catch it before it happens*,* and like that. (Nurse 12).*

#### Sub-theme 2: Positive work environment

The participant clarified that the leaders create a positive work environment by providing easy access for nurses to communicate concerns and ensure a stress-free work environment.

*The managers are making sure their nurses are doing their work good*,* and free from stress*,* making a good environment for the nurses*,* with easy access to the manager*,* and to the head nurse. (Nurse 14).*

#### Sub-theme 3: Reducing stress and workload pressure

The participants noted that managers play a role in reducing stress and workload pressure to maintain high safety standards by monitoring staff well-being.

*The managers are easy to talk to about any situation I have or anything. This will make the load and the stress less on the nurses*,* so they will be doing their job without any pressure on them*,* so it will be free of mistake. (Nurse 12).*

#### Sub-theme 3: Rewards

The participants noted that the hospitals managers reward the units with fewer reported incidents, reinforcing safety-conscious behaviour.

*The teamwork is good*,* the management is good. So the hospital always making a rewards for us. (Nurse 13).*

#### Sub- theme 4: Provision of necessary equipment and resources

Additionally, the participants explained that hospital managers and head nurses contribute to neonatal safety by providing necessary equipment and resources that enhance patient safety.

*Some say we are with you*,* and equipment and everything available to improve the safety. (Nurse 3).*

#### Sub-theme 5: Providing lectures and implementing safety protocols

Hospital managers and head nurses contribute to neonatal safety by conducting patient safety lectures and implementing safety protocols.

*By conducting safety lectures*,* we are taking the educational activities regarding safety because the lectures improve the quality of care and safety. (Nurse 3).*

### Main theme 8: Handoffs and transitions

#### Sub-theme 1: Double-checking protocols

The participant noted that neonatal safety can be compromised during shift changes, admissions, and inter-ward transfers. Therefore, double-checking protocols will be beneficial. Nurses check medications, patient identity, and overall condition before and after transfers to ensure continuity of care.

*We are checking with another nurse. Double check everything from the medication*,* the patient’s side*,* the patient itself from head to toe*,* even inside the unit*,* and by shifting or to shift to another unit. (Nurse 5).*

#### Sub-theme 2: Transferring system

The participant mentioned that strict safety measures are followed during admissions and transfers **(**e.g., babies are placed in incubators or cots rather than being carried by hand). Additionally, a tracking system is in place to ensure the safe movement of neonates within the hospital.

*Always with the admission. For safety*,* we are admitting the babies to the incubator. That means it is fully covered*,* and they always close the door even during the transfer. Also*,* we will shift the baby to the cot. Not in the hand. And we have a tracking system also. (Nurse 3).*

### Main theme 9: Manager expectations and actions promoting patient safety

#### Sub-theme 1: Education and training

The participants clarified that education about safety for new healthcare providers is a strategy that applied to improve patient safety.

*We are doing all the safety and education for the new nurses*,* new doctors*,* whoever is visiting*,* even parents. (Nurse 2).*

#### Sub-theme 2: High expectations

The participants mentioned that the managers expect staff to follow hospital policies and provide safe care.

*They are expecting us to abide by the rules*,* policies*,* and procedures in the hospital. So with this*,* we are guided to meet the expectations they want from us. (Nurse 10).*

### Main theme 10: Improvement strategies

#### Sub-theme 1: Manager pushing

The participants noted that the managers, such as the hospital leaders and the head nurses, play an active role in ensuring neonatal safety by pushing the staff to give high-quality care.

*Yes*,* the managers affect the neonatal safety because they are our managers*,* so they are the ones who will push us to give the best quality for the patient*,* and our patient safety. (Nurse 1).*

#### Sub-theme 2: Team huddles

The participants described that the team influences safety through activities like team huddles.

*Our head nurse is doing huddles every morning. For example*,* the night before*,* we can discuss what the problem is during the night. And then for the incoming day*,* what is the plan for that day*,* so we can prevent errors for their safety. (Nurse 15).*

#### Sub-theme 3: Refresh knowledge and skills

The participant clarified that the staff emphasized the importance of refreshing the knowledge and skills through education.

*And then refresh us always on the proper way to give care and ensure patient safety. So*,* our educators are conducting educational activities every day. (Nurse 15).*

#### Sub-theme 4: Ongoing competency training

The participants mentioned another shared strategy, which is the regular provision of competency refreshers, training sessions, and awareness classes. These sessions are designed to ensure nurses stay up-to-date on protocols and best practices and are confident in performing critical procedures.

*Also*,* given classes and recalling the competency again for all of the staff*,* so we can recall again. (Nurse 15).*

#### Sub-theme 5: Involving all staff in safety and quality projects

The participants noted that the involvement of all staff members, from junior nurses to senior staff, in quality improvement projects, safety discussions, and incident analysis is another common theme. This inclusive approach encourages a collective sense of responsibility and ensures that every team member is aware of safety priorities.

*All the staff… even junior*,* even senior*,* anyone working with the patient directly*,* have to be involved in any single project. (Nurse 15).*

#### Sub-theme 6: Policy reading

The participants emphasized the importance of refreshing their knowledge and skills and adhering to protocols and procedures to protect patients.

*Usually they are doing policy reading in our unit*,* So we are aware of refreshing our skills and knowledge. (Nurse 5).*

#### Sub-theme 7: Education, teamwork, and experience

The participants clarified that education, training, and **t**eamwork are strategies to improve patient safety. Furthermore, experience and continuous practice lead to safety enhancement.

*Education*,* teamwork*,* practice*,* and the more experience*,* the more education experience*,* and the higher education. (Nurse 4).*

#### Sub-theme 8: Scheduling by the managers

The participants noted that managers are responsible for creating staff schedules. Poor scheduling, such as excessive workloads, can lead to burnout and then negatively impact patient safety.

*Managers’ scheduling can affect neonatal safety because they are responsible for giving us the department’s schedule. For example*,* if they give us a full load of duties*,* it will cause burnout of the staff*,* which will impact patient safety. (Nurse 6).*

## Discussion

This study explored perspectives of neonatal nurses on the safety culture concept and the dimensions of patient safety culture that influence patient safety, gathering insights on nurse experiences relating to patient safety issues, and explaining strategies to enhance the patient safety culture. Our results show that patient safety is a structure of both professional and organizational that helps neonatal nurses introduce activities that lead to a lower occurrence of harmful effects, risks, and errors. This is done to enhance the safety of the culture, processes, procedures, behaviours, resources, and environment in health organizations.

The findings is in line with another study emphasized a significant definition of patient safety culture; safety is seen as an all-encompassing concept that includes infection control, alertness, continuous care, careful handling, and protocol adherence because the particular nature of care in NICU due to the high sensitivity and physical differences of newborns, along with the concept that mothers and neonates are a unified care unit, distinguishes the safety culture in the NICU from that of other units [37, 38].

In the present study, the neonatal safety culture is achieved through professional and organizational development. According to professional development, based on the link between interprofessional teamwork and patient safety, we advocate implementing team interventions because professional group, time of employment, and work experience impact perceptions of interprofessional collaboration and patient safety [39]. Therefore, a teamwork atmosphere was linked to lower patient harm and severity-adjusted death [39]. Moreover, participants confirmed that effective cooperation, handoffs, and communication openness among staff are essential measures for improving patient safety incident response. Accordingly, handoff and transitions were considered a key part of patient care, ensuring patient safety and continuity of treatment, and hence optimizing patient outcomes by enhancing medical care quality, lowering the incidence of adverse events [40]. Therefore, creating a culture that promotes error response through teamwork, communication, and handoffs gives healthcare staff the opportunity to learn and improve patient outcomes [40].

Additionally, the primary findings showed that effective information transfer, responsibility, and accountability were required for good impressions of patient safety [41]. Moreover, handoff and transitions were considered a key part of patient care, ensuring patient safety and continuity of treatment, and hence optimizing patient outcomes [41].

As a result, feedback and communication about errors were positively related to patient information transfer. Collaboration in specific units and the frequency of occurrences reported were strongly correlated with personal responsibility transfer during shift changes. Finally, collaboration across units was positively related to unit transfers of patient accountability [41].

Additionally, according to the managerial dimension, the participants explained the importance of hospital leadership, in which leader roles should involve regular rounds, checks on drugs, documentation, and commitment to safety regulations. They promote a pleasant work atmosphere by making it easy for nurses to discuss issues, ensuring a stress-free workplace as mentioned in a previous study that concluded that hospital managers should collaborate with frontline workers to identify and address sources of errors, such as operational failures in nursing units, because improvements to hospital work environments may lower the number of errors and operational failures [42].

On the other hand, management might provide the groundwork for improving patient safety culture and reducing adverse occurrences by implementing various measures such as encouraging adverse event reporting and hosting nurse training courses [43]. Therefore, educational interventions, such as workshops and self-learning, promote patient safety culture [44].

Furthermore, previous studies have provided strong empirical evidence that management safety commitment is associated with patient safety culture [45]. To develop a healthy safety culture, managers must show a visible commitment to patient safety and act as role models [45]. In addition, the participants proposed that nursing managers and administrators should take steps to reduce nursing job stress to a minimum and improve their work environment as illustrated in a previous systematic review in order to offer the best valuable patient care [46]. Therefore, in the context of the current study, participants stated that managers are accountable for designing staff schedules, since bad scheduling, such as an excessive workload, can lead to burnout and have a detrimental impact on patient safety. Previous research found that high workloads among nurses, midwives, and optometrists contributed to burnout, which had a negative impact on patient care, job satisfaction, and personal well-being [47].

Moreover, the participants explained that hospital managers contribute to neonatal safety by providing necessary equipment and resources that enhance patient safety. These resources such as medical equipment, supplies, and technologies, play an important role in healthcare delivery, and their performance can have an impact on patient safety [48].

Otherwise, nurses who have worked under a toxic nurse manager report an increase in adverse events and a lower quality of care in the unit [49]. Therefore, the findings underline that leadership improves nurse motivation, while a clear reward system increases motivation and reinforces the patient safety culture. A previous study supported that a safe culture can be established by reward systems and motivation from leadership [50]. To ensure high-quality healthcare services, practical solutions include enhancing nurse motivation, optimizing reward programs, and strengthening leadership competencies [50].

Regarding the importance of organizational learning, there is a strong positive association between organizational learning and patient safety culture in hospitals. Several organizational learning characteristics were shown to be significantly related to the various components of the pharmacy patient safety culture. The most important parts of patient safety culture were training management that reinforces learning and a supportive learning environment [51].

Our study showed that nonpunitive reports and learning from errors help identify variables that contribute to safety problems. Therefore, reporting systems should prioritize patient outcomes and learn from system issues rather than blaming people [52]. Otherwise, Punitive reports have significant consequences for reporting systems because they may indicate a blame culture and failure to understand systemic influences on behaviour [52].

In addition, Khater et al. [53] found a positive correlation between non-punitive responses to medical errors and the frequency of medical error reporting. As a result, there were fewer PS-related adverse events and patient complaints. Senior nurses’ overall perception was 51.5% before education and 60.6% post-education. The frequency of event reporting increased from 54.2% to 64.3% after applying appropriate educational training.

At the level of organization, the participants ensured that fatigue was frequently linked to mental health issues, reduced nursing performance, and sick leave [16]. This aligned with many previous studies that have demonstrated that nursing fatigue and burnout are connected with bad outcomes for nurses, patient safety, and organizations [16, 54]. Moreover, increased nursing workload leads to a higher risk of nosocomial infections and increased mortality rates [54]. Therefore, the healthcare organization should hire a sufficient number of staff because nurse staffing had varying effects on patient safety, such as the rates of falls and hospital-acquired pressure ulcers, which were positively connected with nurse staffing [55].

Lastely according to strategies to improve patient safety, the current study suggested that education, training, and **t**eamwork are good strategies to accomplish this. Furthermore, experience and continuous practice lead to safety enhancement. So far, Hospitals can drastically minimize medical errors and adverse events by implementing the training program and educating programmers to emphasize patient safety [56, 57].

Other strategy emphasized by the participants was the importance of refreshing their knowledge and skills and adhering to protocols and procedures to protect patients. Therefore, prioritizing patient safety and excellent care is crucial while implementing policies [58].

Moreover, implementing daily huddles could increase medical safety work, problem detection and resolution, situation awareness and teamwork, collaboration and communication across professionals and departments, and patient safety [59].

The limitations of this study were the sample recruited from a single hospital setting as well as a lack of broader demographic diversity. However, this qualitative study explored the perspectives and experiences of nursing managers and nursing frontline staff in NICU, who offer valuable insights into how specific factors can influence safety culture, thereby complementing the findings of previous studies and adding to the existing literature on the topic of patient safety culture. There’s a notable research gap which as qualitative studies focusing on PSC within NICUs are rare, as well as an understanding of how nurses perceive the safety culture within healthcare organizations is still not well-defined [29]. Therefore, this study presents a clear and valuable opportunity—both to contribute original insights and to help shape safety interventions tailored to the complexities of NICU environments in the global and Saudi context. Furthermore, the findings can provide actionable recommendations applicable to NICU context.

## Strengths and limitations of the study

This qualitative study explored the experiences of managers and frontline staff, who offer valuable insights into how specific factors such as effective team communication, adequate staffing, effective leadership, and collaboration can influence safety culture, thereby complementing the findings of previous studies and adds to the existing literature on the topic of patient safety culture. The current study holds particular relevance in Saudi Arabia, where the healthcare industry is experiencing major changes aimed at achieving the high health standards outlined in Saudi Vision 2030. By exploring the patient safety culture and dimensions within healthcare environments in NICU that shape nurses’ perceptions—such as leadership structures, communication systems, and policy frameworks—the research will yield significant insights. Moreover, these results will assist healthcare leaders and policymakers in crafting and executing culturally appropriate reforms that strengthen the culture of patient safety, enhance the quality and safety of healthcare, and further the country’s objective of becoming a frontrunner in healthcare innovation, particularly in NICUs.

However, this study has limitations. It was conducted within a single hospital setting as well as a lack of broader demographic diversity. Therefore, these findings may not be transferable to other health organizations. Future research must be conducted in a broader setting and demographic diversity.

## Conclusion

This study provides an in-depth exploration of how neonatal nurses perceive patient safety culture within the unique, high-risk NICU atmosphere that highlights unit-specific challenges such as premature infant fragility. In addition, captures the lived experiences, beliefs, and values that shape how nurses understand and enact safety practices as well as reveals unspoken norms, informal practices, and power dynamics that influence safety but are not easily measurable in surveys.

The findings revealed systemic barriers (staffing shortages, workload, communication gaps) and facilitators (teamwork, leadership support, peer mentoring). Furthermore, brings out emotional and ethical dimensions of safety such as fear of blame, moral distress when unable to deliver optimal care). Moreover, offers practical recommendations for tailored interventions in NICUs that involve education, training, teamwork, implementing daily huddles, continuous practice, encouraging open reporting and non-punitive safety climates, all of theses lead to safety enhancement. Therefore, this study contributes to the improvement of current safety culture frameworks by adding insights unique to neonates and encourages greater staff engagement in safety improvement initiatives.

Future research should explore how these interventions can further enhance patient safety culture in NICUs.

## Data Availability

The dataset analyzed during the current study are available from the corresponding author on reasonable request.

## Declarations

### Declarations

Data sources for this study were performed in accordance with relevant guidelines and regulations.

## Abbreviations

PSC: Patient Safety Culture
NICU: Neonatal Intensive Care Unit
WHO: World Health Organization
HSOPSC: Hospital Survey on Patient Safety Culture
PICUs: Paediatric Intensive Care Units
AHRQ: Agency for Healthcare Research and Quality
ICN: International Council of Nursing
MOH: Ministry of Health
